# Electronic structure engineering of phosphorene through transition metal functionalization for toxic gas detection

**DOI:** 10.1039/d6ra00934d

**Published:** 2026-05-01

**Authors:** Shah Nawaz Khan, Akbar Hussain, Shabeer Ahmad Mian, Joonkyung Jang

**Affiliations:** a Department of Physics, University of Peshawar Peshawar Pakistan shabeerahmad@uop.edu.pk; b Department of Nano Energy Engineering, Pusan National University Busan Republic of Korea jkjang@pusan.ac.kr

## Abstract

Designing sensitive, selective, and recyclable materials for toxic gas detection is crucial for environmental monitoring and public safety. In this study, density functional theory calculations are employed to systematically investigate the adsorption behavior, electronic structure modulation, charge transfer, optical response, and sensing performance of CO, CO_2_, H_2_S, and SO_2_ on cobalt-doped and cobalt-adsorbed phosphorene. Cobalt doping transforms pristine phosphorene from a direct-bandgap semiconductor (1.06 eV) into an indirect-bandgap semiconductor with a reduced bandgap of 0.83 eV, while cobalt adsorption further narrows the bandgap to 0.42 eV. Across all target gases and studied temperatures, the Co-doped configuration exhibits stronger adsorption and moderate charge transfer, which govern the observed bandgap modulation and sensing response, while the Co-adsorbed system shows excessive charge transfer, leading to near-irreversible adsorption. Among the investigated gases, SO_2_ and CO show the highest sensing responses, whereas CO_2_ interacts weakly with both surfaces. Based on literature trends, Co-modified phosphorene is expected to exhibit selective responses toward other common gases, such as NH_3_ and NO_2_, with minimal interference from H_2_O. Recovery-time analysis indicates rapid and reversible desorption under visible-light irradiation for the Co-doped system. In contrast, the Co-adsorbed system exhibits pronounced charge transfer, leading to strongly bound adsorption that is nearly irreversible. These results suggest that Co-doped phosphorene is a promising candidate for gas sensing based on computational predictions.

## Abstract 2

Cobalt doping enables reversible sensing, while adsorption causes near-irreversible binding for gas capture. SO_2_ and CO show strong responses, while CO_2_ is weak. Performance improves *via* light recovery, bandgap tuning, and stability.

## Graphical abstract

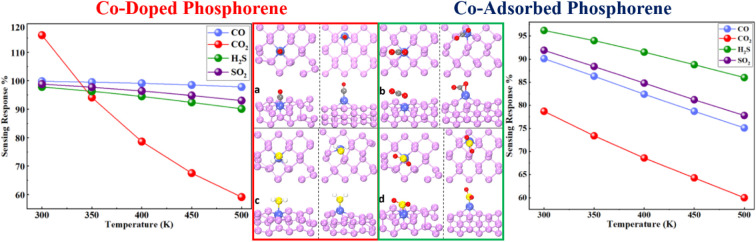

## Introduction

Industries, power plants, and vehicles emit substantial quantities of toxic gases into the atmosphere due to the use of fossil fuels as energy sources.^1,2^ The release of large volumes of toxic gases, such as CO_2_, H_2_S, SO_2_, NH_3_, CO, NO, and NO_2_, contributes to air pollution and poses serious risks to public health.^3–5^ The World Health Organization has identified air pollution as a “silent killer” responsible for approximately seven million deaths annually, including thousands of children.^6,7^ Such mortalities are predominantly associated with respiratory pathologies and cardiovascular conditions, including myocardial infarction, stroke, and lung cancer.^8^ Therefore, the detection of toxic gases is critical for public safety, environmental monitoring, medical diagnostics, agricultural practices, and industrial chemical processes.^9^ For many industries, identifying hazardous gases, such as NH_3_ and CO, is essential to ensure compliance with environmental standards and mitigate their harmful impacts.^10–12^ Recently, gas sensors have become indispensable for applications across multiple domains.^13^ An ideal gas sensor exhibits characteristics such as high selectivity, high sensitivity, fast recovery time, high precision, high stability, low hysteresis, high repeatability, and high resolution.^14,15^ Proper material selection is therefore essential to ensure effective gas-sensing performance.^16^ Materials with two-dimensional (2D) structures, such as graphene, molybdenum disulfide, and other transition metal dichalcogenides (TMDs), have drawn considerable attention from scientists because of their excellent electrical, thermal, and mechanical properties.^17,18^ These materials offer a high surface-to-volume ratio, high carrier mobility, and advanced synthesis possibilities compared to their one-dimensional (1D) and bulk counterparts.^19–21^ In addition, 2D materials exhibit significant potential for applications in photovoltaics, photodetectors, nanoelectronics, light emitters, drug delivery, and gas sensors.^22^ Graphene possesses exceptional electron mobility and a high surface area relative to its volume, making it well-suited for use in energy-storage devices and gas monitoring.^23^ However, π–π electron coupling in graphene slows charge transfer and reduces sensitivity to gas molecules. Additionally, the zero-bandgap nature of graphene limits its applicability in gas sensors. Consequently, exploring 2D materials with tunable bandgaps and enhanced gas-sensing capabilities remains an important research direction, and appropriately engineered bandgaps constitute a critical area of ongoing research.^24^ In 2014, phosphorene was successfully fabricated from bulk black phosphorus using mechanical exfoliation.^25^ Phosphorene, a single atomic layer derived from black phosphorus, is one of the four known allotropes of phosphorus.^26^ It is a novel 2D semiconductor material characterized by a puckered honeycomb structure with an orthorhombic symmetry.^27^ Phosphorene has a direct bandgap that varies with the number of layers, ranging from 0.33 to 1.50 eV.^28–30^ Furthermore, its charge-carrier mobility is approximately 1000 cm^2^ V^−1^ s^−1^, which is higher than that of MoS_2_.^30^ The surface-to-volume ratio of phosphorene is higher than that of other 2D materials, providing distinct advantages for gas-sensing applications.^31^ Phosphorene finds diverse applications in batteries, transistors, solar cells, drug-delivery systems, quantum dot synthesis, and gas sensing.^32^ Gas molecules generally exhibit higher adsorption energies on phosphorene than on graphene and other 2D materials.^33^ Its high surface-to-volume ratio and strong chemical reactivity make it highly suitable for gas-sensing applications.^34^ Previous studies have demonstrated the potential of phosphorene as an effective gas-sensing material. Despite notable progress, understanding the interactions between toxic gases, volatile organic compounds (VOCs), and phosphorene remains an active area of research. First-principles studies have explored the adsorption characteristics of various gas molecules on phosphorene, including rare gases,^35^ methane on doped systems,^30^ and toxic species such as phosgene.^36^ The effect of metal-atom substitution on CO adsorption has also been investigated,^37^ while pristine phosphorene has been proposed as a promising gas sensor for the selective detection of SF_6_ decomposition products.^38^ In addition, the sensitivity of phosphorene has been widely reported, although the recovery dynamics remain less explored,^39^ and fundamental studies on physisorption have established a baseline for small-molecule interactions.^40^ In addition, the role of metal adatoms has been examined. However, these studies are generally limited to single gas molecules or a single type of surface modification, and even in multi-gas investigations, different functionalization strategies are rarely compared under identical computational conditions. For instance, Lei S. *et al.*^43^ and Sun X. *et al.*^37^ focused on CO adsorption using substitutional doping, while Kou L. *et al.*^39^ considered pristine phosphorene; Cai Y. *et al.*^40^ examined physisorption without functionalization. As a result, a consistent understanding of how different modification approaches influence sensing behavior is still lacking.

In this work, a systematic comparison between Co-doped and Co-adsorbed phosphorene is performed for multiple toxic gases (CO, CO_2_, H_2_S, and SO_2_) within a unified DFT framework. These gases are common atmospheric pollutants and have been frequently investigated in previous studies on phosphorene and other 2D materials.^41,42^ This direct, side-by-side comparison of substitutional doping and adatom adsorption under identical conditions has not been systematically addressed in previous studies. Such an approach enables clear identification of how the mode of cobalt incorporation governs adsorption strength, charge transfer, and electronic response. Furthermore, beyond adsorption energetics, this study integrates electronic structure, optical response, charge transfer, sensing characteristics, and recovery behavior within a single framework, allowing the simultaneous evaluation of sensitivity, selectivity, and reversibility. Therefore, the novelty of this work lies not merely in extending the range of gases but in establishing a comparative and mechanistic understanding of cobalt-modified phosphorene, providing more reliable design guidelines for high-performance gas sensors.

## Computational methods and material parameters

Co-doped and Co-adsorbed phosphorene monolayers were investigated for the adsorption of different toxic gases using DFT simulations.^43^ For each gas molecule, several initial adsorption configurations, including different orientations and adsorption sites, were considered. Full structural optimization was performed, and the most stable configuration with the lowest total energy was selected for further analysis. The adsorption and sensing of toxic gases were examined using the Spanish Initiative for Electronic Simulations with Thousands of Atoms (SIESTA) computational method.^14^ A 3 × 2 phosphorene supercell containing 24 P atoms was constructed in Materials Studio, with lattice parameters of *a* = 3.63 Å and *b* = 4.29 Å.

A 12 Å vacuum slab was introduced to eliminate periodic interactions.^44^ The generalized gradient approximation (GGA) with the revised Perdew–Burke–Ernzerhof (RPBE) exchange–correlation functional was employed for structural optimization. Geometry optimizations were performed using Monkhorst–Pack *k*-point meshes of 3 × 3 × 1 and 6 × 6 × 1. A real-space grid cutoff of 198 Ry (≈2.7 keV electron kinetic energy) was used in the SIESTA calculations. The computational setup employed norm-conserving pseudopotentials in a fully nonlocal semicore form and a double-zeta basis set to describe the valence orbitals. The computational parameters, including *k*-point sampling, energy cutoff, and supercell size, were selected based on well-established convergence-tested settings reported in previous phosphorene-based studies, ensuring reliable adsorption energies and electronic properties.^45–47^

The 3 × 2 supercell provides sufficient separation between periodic images, minimizing artificial interactions between Co atoms and adsorbed gas molecules. The Co concentration (∼4 at%) was chosen to represent experimentally relevant doping levels while avoiding significant Co–Co interactions. Such doping levels are feasible in two-dimensional materials and can be achieved *via* substitutional incorporation during growth (*e.g.*, chemical vapor deposition) or post-synthetic functionalization, including adsorption, followed by annealing or defect-assisted doping. Pristine phosphorene is known to degrade under ambient conditions due to oxidation in the presence of oxygen and moisture; previous studies have shown that transition metal doping and surface functionalization can enhance its chemical stability. Additionally, encapsulation techniques have been demonstrated to significantly improve its environmental stability. Therefore, the chosen model is both computationally practical and experimentally relevant under controlled conditions.^39,48^

All calculations in this work were performed using a non-spin-polarized approach. While cobalt is a magnetic transition metal and its spin polarization may influence the electronic structure through Co 3d states, previous studies on transition-metal-doped phosphorene have shown that key properties, such as adsorption energies, charge transfer, and relative sensing trends, remain qualitatively consistent.^14,43^ Therefore, the non-spin-polarized framework adopted here is sufficient for comparative analysis. However, spin-polarized calculations could further refine the understanding of magnetic interactions and will be explored in future investigations.

## Calculation of adsorption energy

Determining the adsorption energy is essential for understanding the interaction mechanisms of toxic gases with phosphorene. The adsorption energy provides insight into the interaction strength, thermal stability, and the feasibility of experimental realization. In this study, the interaction energies of gas molecules on Co-doped and Co-adsorbed phosphorene were computed using eqn (1) and (2).^30,35,49–51^

1 *E*^Co-doped^_ads_ = *E*_(Co-doped+gas)_ − *E*_Co-doped_ − *E*_gas_

2 *E*^Co-adsorbed^_ads_ = *E*_(Co-adsorbed+gas)_ − *E*_Co-adsorbed_ − *E*_gas_

In eqn (1), *E*^Co-doped^_ads_ represents the adsorption energy of the Co-doped system and *E*_(Co-doped+gas)_, *E*_Co-doped_, and *E*_gas_ correspond to the total energies of the gas-adsorbed doped system, the isolated Co-doped phosphorene, and the isolated gas molecule, respectively. Similarly, in eqn (2), *E*^Co-adsorbed^_ads_ denotes the adsorption energy of the Co-adsorbed system and *E*_(Co-adsorbed+gas)_, *E*_Co-adsorbed_, and *E*_gas_ represent the total energies of the gas-adsorbed Co-adsorbed system, the isolated Co-adsorbed phosphorene, and the isolated gas molecule, respectively. All adsorption energies reported in the tables and text have been carefully checked and are consistently reported using the negative sign convention, where a negative adsorption energy indicates exothermic (thermodynamically favorable) adsorption.^51^

## Results and discussion

### Pristine, Co-doped, and Co-adsorbed phosphorene structures

**Fig. 1:**
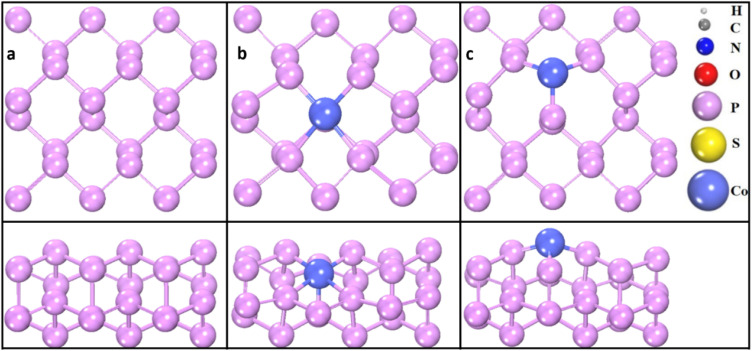
Top and side views of the phosphorene-based systems: (a) pristine phosphorene, (b) Co-doped phosphorene and (c) Co-adsorbed phosphorene. Phosphorus atoms are shown in orange and cobalt atoms in blue. Bond lengths are indicated in Å. The figure highlights the structural modifications due to Co doping and adsorption.

In the optimized phosphorene structure, each phosphorus atom is covalently bonded to three adjacent phosphorus atoms, resulting in a puckered, honeycomb-like framework similar to graphene but with intrinsic wrinkles.^52^ Upon the substitution of a Co atom for a phosphorus atom, the system fully relaxes. The doping concentration of a single Co atom in a phosphorene monolayer supercell containing 24 phosphorus atoms corresponds to approximately 4%. For Co adsorption on phosphorene, two main adsorption sites are possible: directly above a phosphorus atom or at the hollow site of the honeycomb structure, approximately 2.60 Å above the center. The hollow site is energetically more favorable because it allows interactions with three neighboring phosphorus atoms. This substitution and adsorption significantly affect the overall crystal lattice, inducing local deformations in bond lengths and bond angles, which are influenced by the presence of the dopant. All geometries reached their respective minimum energy states during optimization, indicating structural stability. Table 1 presents the calculated bond lengths and angles obtained from the relaxed structures of the pristine, Co-doped, and Co-adsorbed phosphorene (Fig. 1).

**Table 1:** Bond lengths (*d*_1_ and *d*_2_) and bond angles (*θ*_1_ and *θ*_2_) of the pristine, Co-doped, and Co-adsorbed phosphorene

Dopants	*d*_1_ (Å)	*d*_2_ (Å)	*θ*_1_ (°)	*θ*_2_ (°)
Pristine (this work)	2.281	2.263	101.9	95.6
Pristine^14^	2.262	2.241	101.6	96.6
Co-doped (this work)	2.207	2.233	94.1	85.5
Co-doped^43^	—	2.232	—	—
Co-adsorbed	2.180	2.257	113.8	117.4

In the pristine phosphorene, the P–P bond lengths *d*_1_ and *d*_2_ are 2.280 Å along the armchair direction and 2.263 Å along the zigzag direction, respectively, while the corresponding bond angles *θ*_1_ and *θ*_2_ are 101.0° and 95.6°, respectively. For the Co-doped phosphorene, the P–P bond lengths are reduced to 2.207 Å in the armchair direction and slightly increased to 2.233 Å in the zigzag direction, whereas the bond angles *θ*_1_ and *θ*_2_ decrease to 94.1° and 85.5°, respectively. In the Co-adsorbed structure, the Co–P bond length is 2.098 Å, and the P–P bond length is 2.257 Å. The corresponding bond angles *θ*_1_ and *θ*_2_ increase significantly to 113.8° and 117.4°, respectively.

**Fig. 2:**
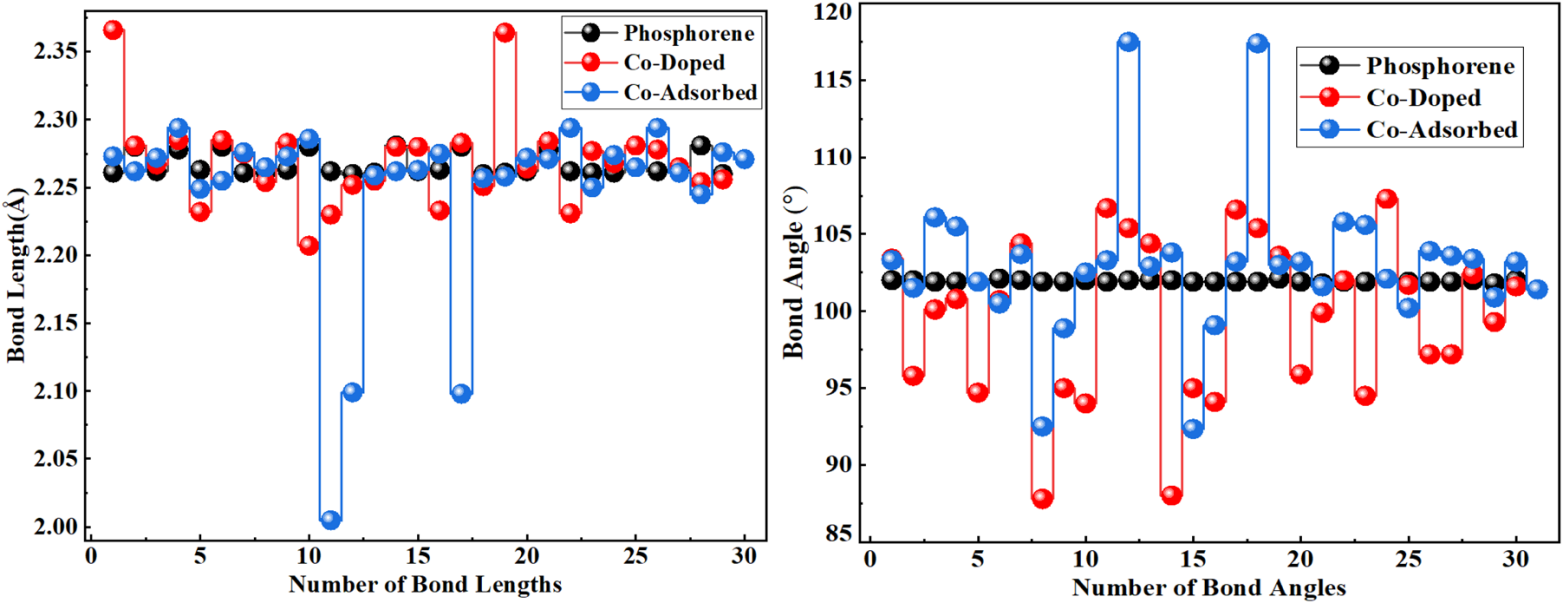
Comparison of the structural parameters of the pristine, Co-doped, and Co-adsorbed phosphorene. The figure shows variations in bond lengths (*d*_1_ and *d*_2_, in Å) and bond angles (*θ*_1_ and *θ*_2_, in degrees) for the three systems. The results illustrate the structural distortions in the phosphorene lattice induced by Co doping and adsorption.

The bond lengths and angles for the pristine, Co-doped, and Co-adsorbed phosphorene are plotted in Fig. 2. These results are consistent with those reported in the literature.^25,53^ These variations in bond lengths and angles induced by Co doping and adsorption reflect localized geometric distortions in the phosphorene lattice. These distortions enhance surface reactivity and promote the formation of favorable active sites for toxic-gas adsorption.

### Electronic structure and properties

**Fig. 3:**
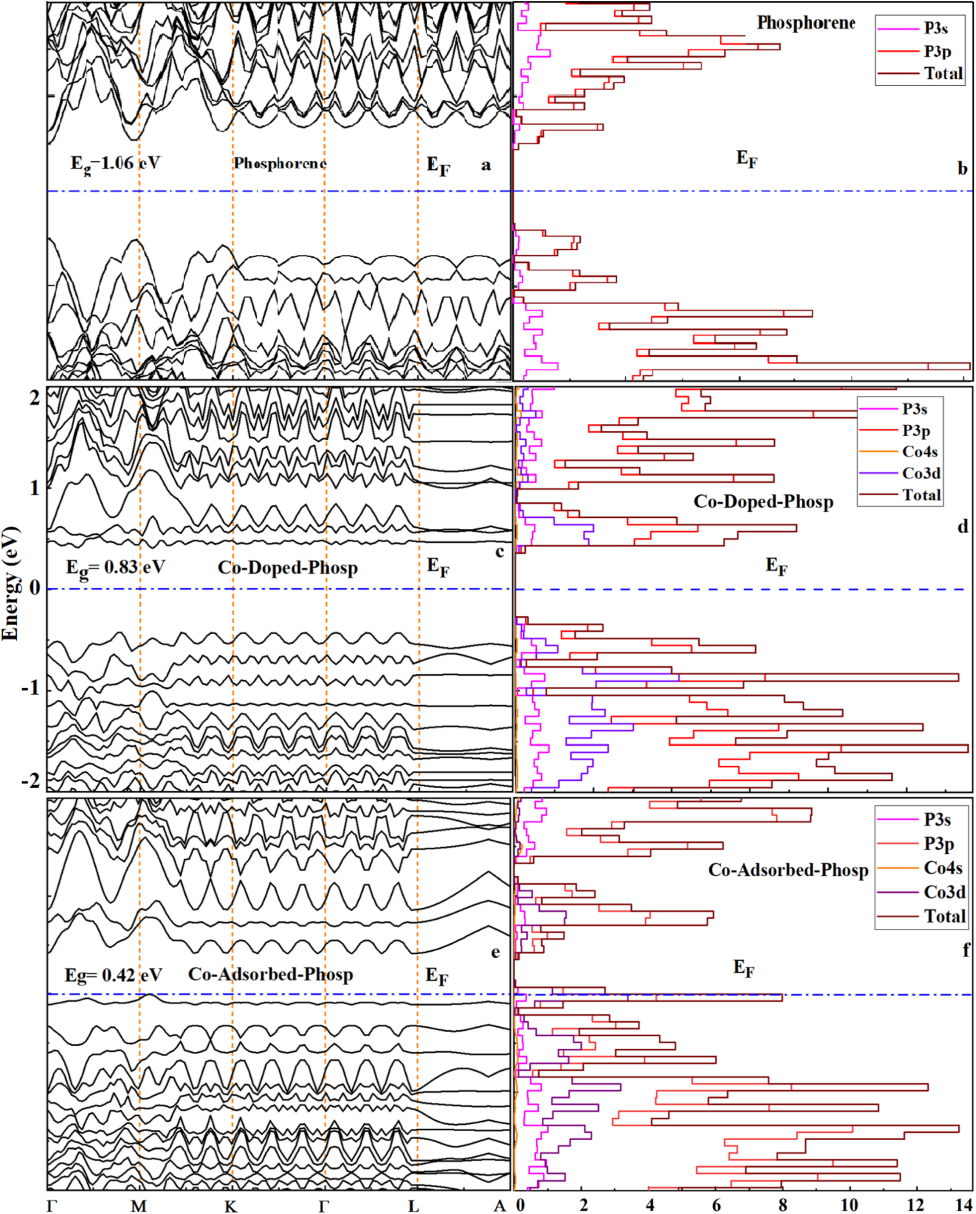
Band structures (left) and projected density of states (PDOS, right) of the (a and b) pristine, (c and d) Co-doped, and (e and f) Co-adsorbed phosphorene. The Fermi level (*E*_F_ = 0 eV) is shown by the dashed blue line. Co incorporation reduces the bandgap from 1.06 eV to 0.83 eV and 0.42 eV.

In the next step, the electronic band structure and PDOS of the pristine, Co-doped, and Co-adsorbed phosphorene systems were plotted to investigate their electronic behavior, and the results are shown in Fig. 3(a–f). As evidenced by the bandgap widths shown in Fig. 3(a), the pristine phosphorene exhibited a direct bandgap of 1.06 eV, which is in good agreement with previously reported values in the literature.^54,55^ The substitution of a Co atom in phosphorene decreases the bandgap from 1.06 eV to 0.83 eV (Fig. 3(c)). This transformation is attributed to the hybridization between the Co 3d orbitals and the P 3p states, which perturbs the band edges and modifies the carrier transport properties owing to the formation of new energy states near the Fermi level. Calculations confirmed that the pristine phosphorene is a direct bandgap material; however, Co doping changes the bandgap from direct to indirect, which strongly influences optical absorption. These results imply that Co doping increases the responsiveness of phosphorene for gas detection, a trend also observed in previous studies.^25,43^ With Co adsorption, the bandgap of the Co-adsorbed system decreases to 0.42 eV (Fig. 3(e)). The PDOS confirmed significant alterations in the electronic states of the doped phosphorene compared to its pristine counterpart. Furthermore, substituting a Co atom into the pristine phosphorene resulted in a notable distortion in the PDOS near the Fermi level. This deformation is attributable to the emergence of an additional electronic band above the Fermi level compared to the pristine phosphorene. Phosphorus 3p states made the most significant contribution to the valence band near the Fermi level. Strong hybridization between the Co 3d and P 3p states introduced additional states, modifying the density of states near the valence region. The Co 3d states contributed significantly to the lower edge of the conduction band. Hybridization between the Co 3d, P 3p, and P 3s states led to the redistribution of electronic states, influencing charge transport. Additional peaks in the PDOS arose because of the strong interactions between these orbitals, which affected the overall electronic structure.

### Optical absorption and conductivity

**Fig. 4:**
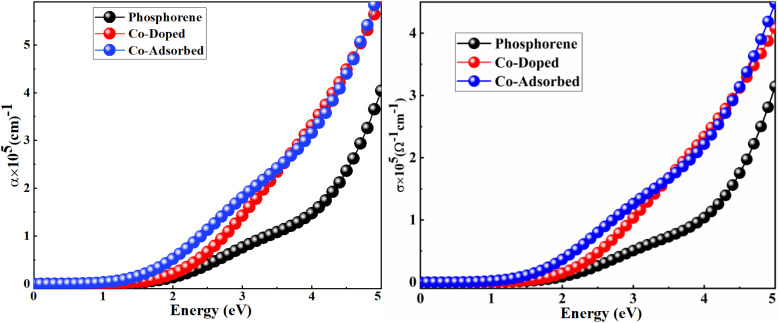
Optical absorption (right) and optical conductivity (left) of the pristine, Co-doped, and Co-adsorbed phosphorene. The *x*-axis represents the photon energy (eV), while the *y*-axis shows the absorption coefficient (*α* × 10^5^ cm^−1^) and optical conductivity (*σ* × 10^5^ Ω^−1^ cm^−1^), respectively. Black, red, and blue lines indicate the pristine, Co-doped, and Co-adsorbed systems, respectively.

The optical absorption spectra of the pristine, Co-doped, and Co-adsorbed phosphorene, with characteristic absorption peaks at 1.06, 0.83, and 0.42 eV, respectively, are presented in Fig. 4. These spectra provide insight into the electronic structure and bandgap features of the material. The high density of states near the Fermi level facilitates electron transitions to the conduction band upon photon absorption, which directly influences the optoelectronic behavior relevant for gas sensing applications. Incorporation of Co into pristine phosphorene enhances the optical absorption and broadens the absorption spectrum. Importantly, the absorption peaks correspond to photon energies capable of promoting electron excitation under visible light. This suggests that light illumination can assist in the desorption of gas molecules from the phosphorene surface, thereby improving sensor recovery and reversibility.

The optical conductivities (*σ*) of the pristine, Co-doped, and Co-adsorbed phosphorene were evaluated using eqn (3) to assess the charge-transport efficiency.^49,56^3
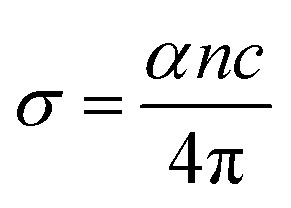
Here, *c*, *n*, and *α* denote the speed of light, refractive index, and absorption coefficient, respectively. According to eqn (3), optical conductivity depends directly on the absorption coefficient, reflecting the material's response to incident photons. As shown in the right panel of Fig. 4, Co incorporation enhances the optical conductivity of phosphorene, indicating more efficient charge transport under illumination. Such an improvement can facilitate electron-transfer processes that promote the light-assisted desorption of adsorbed gas molecules, thereby enhancing the overall performance of phosphorene-based sensors.

Overall, the increased optical absorption and conductivity of the Co-doped and Co-adsorbed phosphorene are not only indicative of improved optoelectronic behavior but also play a crucial role in enabling visible-light-assisted recovery in gas sensing applications.

### Adsorption and electronic properties of gases on the Co-doped and Co-adsorbed phosphorene

**Fig. 5:**
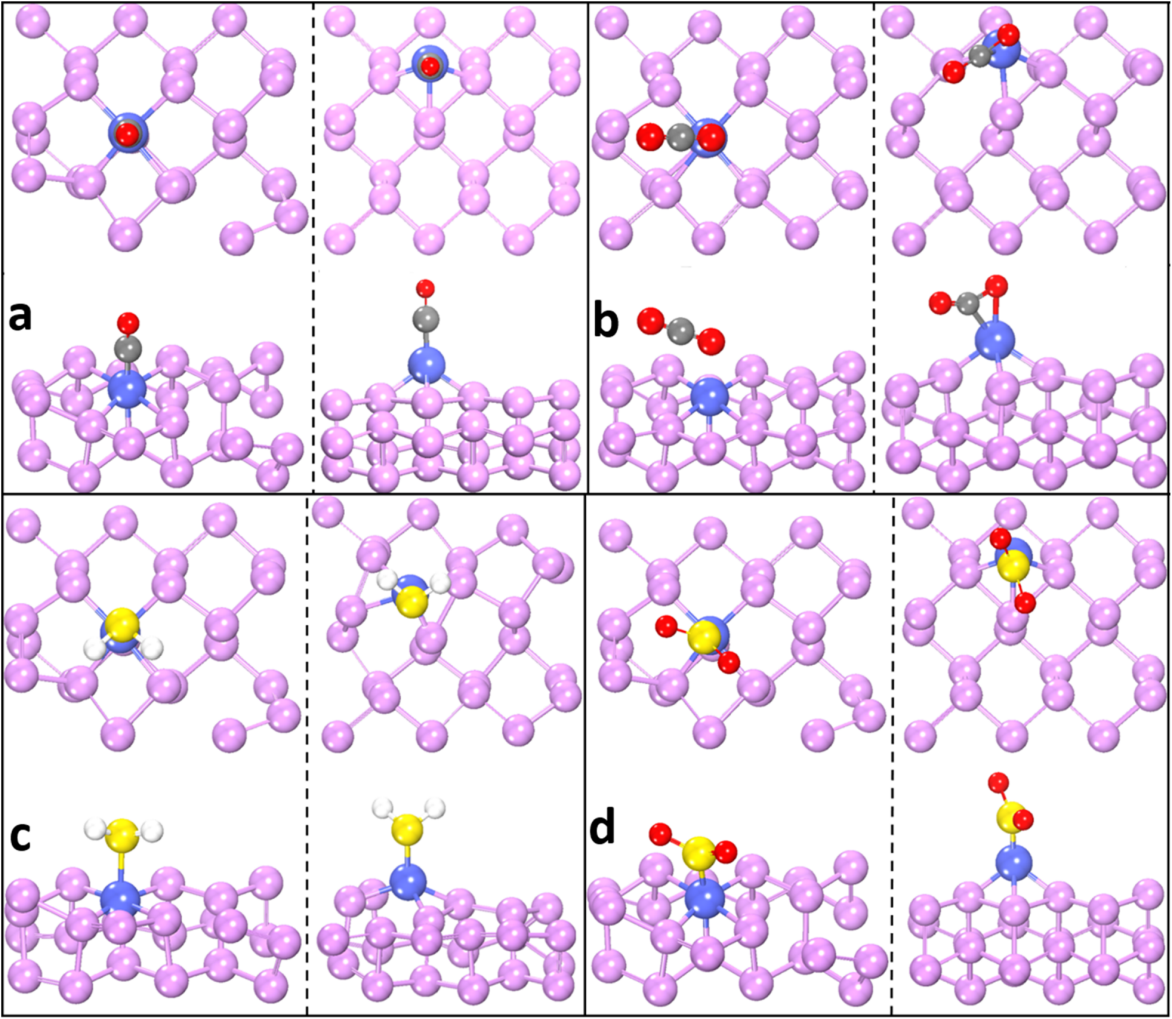
Optimized adsorption configurations of CO, CO_2_, H_2_S, and SO_2_ on phosphorene. Panels (a–d) correspond to CO, CO_2_, H_2_S, and SO_2_, respectively. For each gas, the left panel shows the Co-doped phosphorene, and the right panel shows the Co-adsorbed phosphorene. In each panel, the top row represents the top view, and the bottom row represents the side view of the adsorbed gas molecule. Color scheme: P (purple), Co (blue), O (red), C (gray), S (yellow), and H (white).

**Fig. 6:**
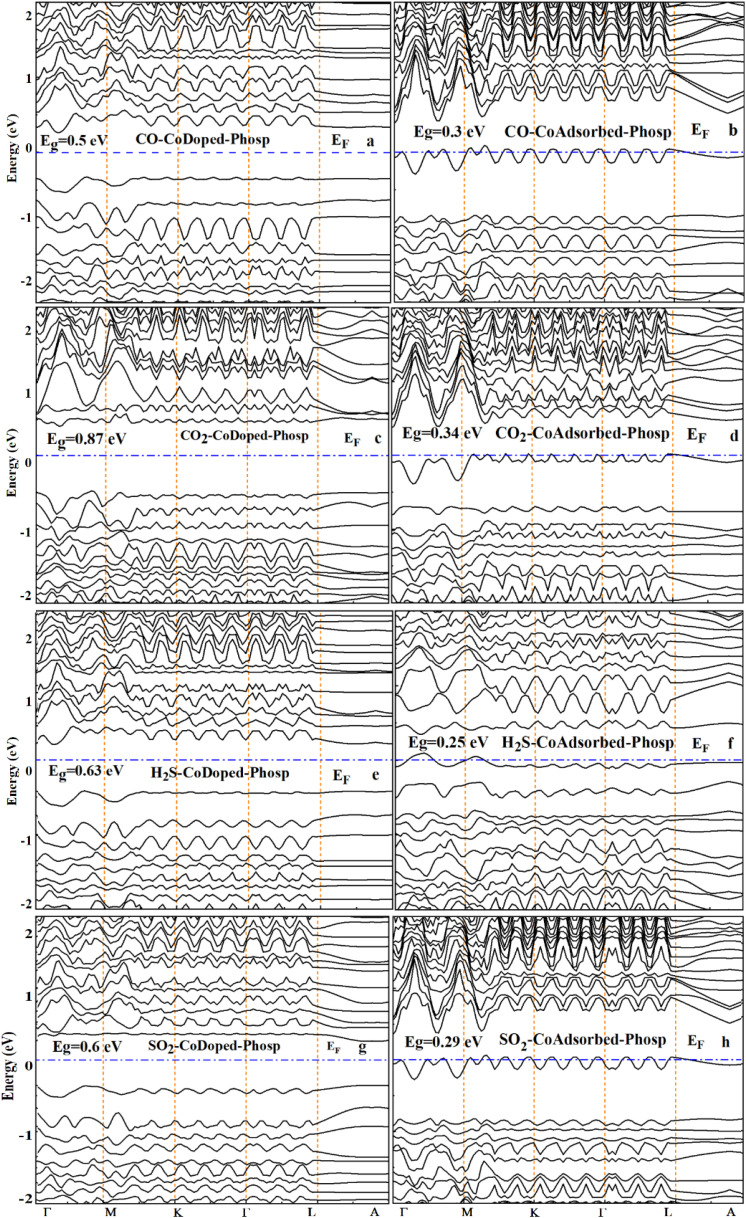
Electronic band structures of the gas-adsorbed Co-modified phosphorene for the CO, CO_2_, H_2_S, and SO_2_. Panels (a, b), (c, d), (e, f), and (g, h) correspond to CO, CO_2_, H_2_S, and SO_2_, respectively. Panels (a, c, e and g) represent the Co-doped phosphorene, while (b, d, f and h) represent the Co-adsorbed phosphorene. The dashed horizontal line indicates the Fermi level (*E*_F_ = 0 eV).

**Fig. 7:**
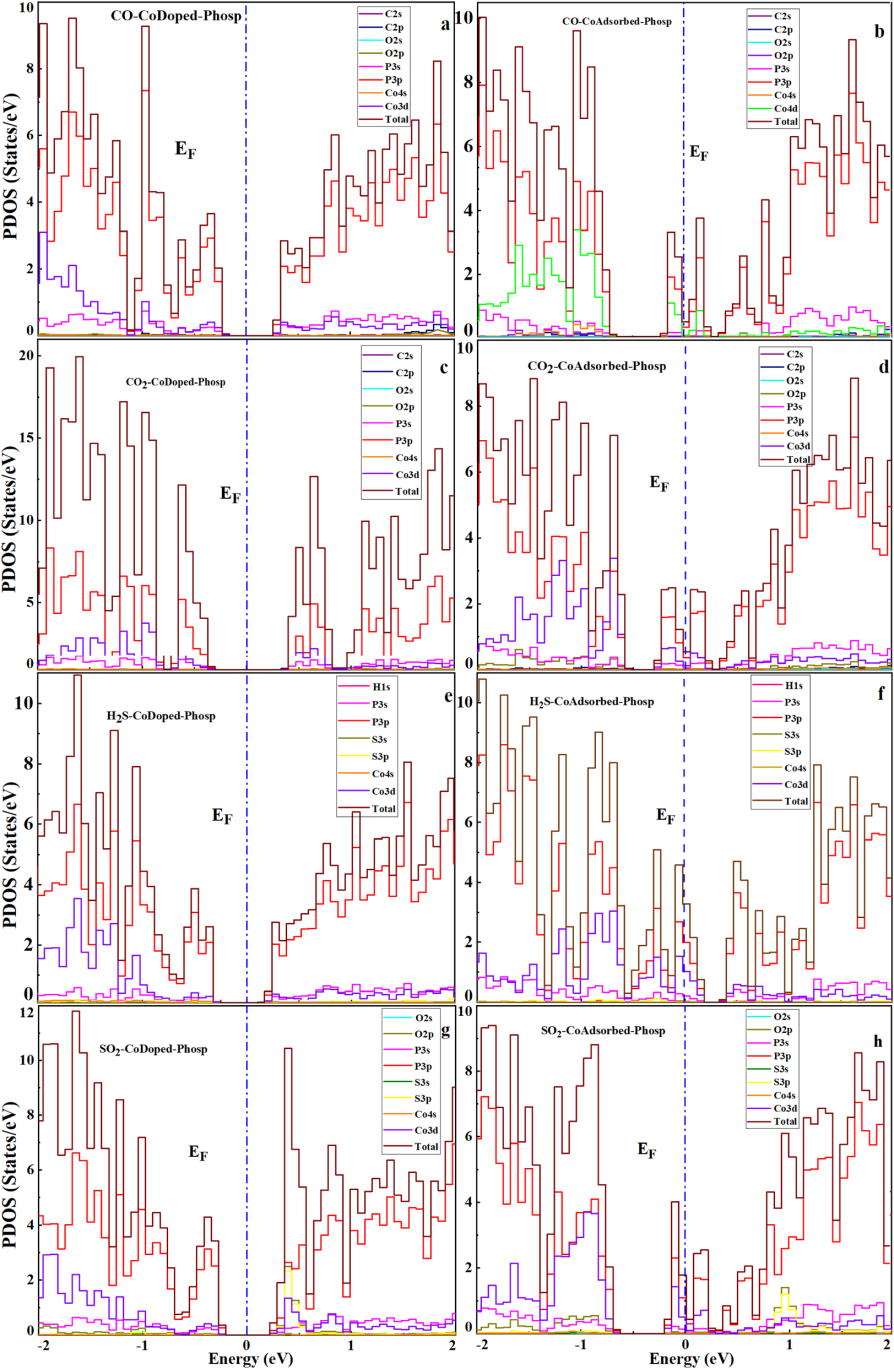
Projected density of states (PDOS) of the gas-adsorbed Co-modified phosphorene for CO, CO_2_, H_2_S, and SO_2_. Panels (a, b), (c, d), (e, f), and (g, h) correspond to the CO, CO_2_, H_2_S, and SO_2_ adsorptions, respectively. Panels (a, c, e and g) represent the Co-doped phosphorene, while panels (b, d, f and h) represent the Co-adsorbed phosphorene. The dashed vertical line indicates the Fermi level (*E*_F_ = 0 eV). The PDOS demonstrates significant hybridization between the Co 3d states and the gas molecule orbitals near the Fermi level, providing insights into the electronic origin of the sensing behavior.

To comprehensively assess the sensing performance of cobalt-modified phosphorene, we conducted a comparative analysis of CO, CO_2_, H_2_S, and SO_2_ on both the Co-doped and Co-adsorbed phosphorene systems. The optimized adsorption configurations are shown in Fig. 5, whereas the corresponding electronic properties, including the band structures and projected density of states (PDOS), are presented in Fig. 6 and 7. Upon adsorption, all gas molecules interact with the Co-modified phosphorene surface, inducing notable changes in both structural and electronic properties. Generally, the distance between the gas molecules and the Co atom decreases after structural optimization, confirming the stability of adsorption. However, the interaction strength varies significantly depending on the type of gas molecule and the modification strategy. Among all the gases, CO exhibits the strongest interaction in both systems. In the Co-doped phosphorene, the distance between the CO molecule and the Co atom decreases from 2.549 Å to 1.779 Å, accompanied by an increase in the Co–P bond lengths and minor variations in bond angles, indicating slight structural deformation. In the Co-adsorbed system, the interaction is even stronger, with the distance further reduced to 1.762 Å and more pronounced changes in the P–P and Co–P bond lengths. This strong interaction is consistent with the calculated adsorption energies of −1.20 eV and −4.00 eV for Co-doped and Co-adsorbed systems, respectively. By contrast, CO_2_ exhibits a relatively weak interaction, particularly in the Co-doped system. The distance between the oxygen atom and Co decreases slightly from 2.808 Å to 2.402 Å, with minimal structural distortion, which corresponds to a low adsorption energy of −0.42 eV. In the Co-adsorbed phosphorene system, however, the interaction strengthens, as evidenced by the reduction in the Co–C and Co–O distances to approximately 2.0 Å and an increased adsorption energy of −1.9 eV. For H_2_S adsorption, a moderate interaction is observed in the Co-doped system, where the S–Co distance decreases from 2.549 Å to 2.310 Å, accompanied by slight changes in bond lengths and angles. In the Co-adsorbed system, adsorption is stronger, with the S–Co distance further reduced to 2.172 Å and a higher adsorption energy of −2.60 eV observed. Similarly, SO_2_ demonstrates a relatively strong interaction with both systems. In Co-doped phosphorene, the S–Co distance decreases from 2.680 Å to 2.129 Å, accompanied by noticeable bond distortions. In the Co-adsorbed system, the interaction is stronger, with a shorter distance of 2.05 Å and more significant structural modifications. The adsorption energy is −0.55 eV for Co-doped and −3.10 eV for Co-adsorbed phosphorene, confirming stronger binding in the latter.

The calculated band structures and projected density of states (PDOS) reveal that gas adsorption significantly alters the bandgap of phosphorene, which is a key parameter governing sensing performance, as it directly affects electrical conductivity. In Co-doped phosphorene, CO adsorption reduces the bandgap from 0.83 eV to 0.50 eV, while H_2_S and SO_2_ lower it to 0.63 eV and 0.60 eV, respectively. By contrast, CO_2_ slightly increases the bandgap to 0.87 eV, reflecting weaker electronic coupling. A more pronounced electronic response is observed in the Co-adsorbed phosphorene system, where the bandgap decreases from 0.42 eV to 0.30 eV (CO), 0.34 eV (CO_2_), 0.25 eV (H_2_S), and 0.29 eV (SO_2_). This consistent bandgap narrowing indicates enhanced electrical conductivity and stronger sensitivity toward gas adsorption. The PDOS results (Fig. 7(a–h)) further support these findings, showing increased hybridization between the orbitals of the gas molecules and the Co atom near the Fermi level. The adsorption energies and corresponding bandgap values are listed in Table 3. This effect is particularly strong for CO, H_2_S, and SO_2_ adsorption, especially in the Co-adsorbed system, indicating stronger electronic coupling. To further clarify the microscopic origin of these electronic changes and their impact on sensing behavior, a Mulliken charge population analysis was performed.

### Charge transfer analysis (Mulliken population analysis)

To elucidate the interaction mechanisms between gas molecules and the Co-modified phosphorene, Mulliken charge population analysis was performed. This method provides quantitative insight into both the direction and magnitude of electron transfer, which is crucial for understanding and optimizing gas-sensing performance. Positive values of charge transfer indicate electron donation from the gas molecule to the phosphorene surface, whereas negative values indicate electron withdrawal from the surface by the molecule. The Mulliken charge transfer (Δ*Q*) is calculated using eqn (4).

4 Δ*Q*_doped_ = *Q*^isolated^_gas_ − *Q*^adsorbed^_gas_

Here, *Q*^isolated^_gas_ represents the Mulliken charge of the isolated gas molecule, while *Q*^adsorbed^_gas_ denotes the Mulliken charge after adsorption on the phosphorene surface. Positive Δ*Q* values indicate electron donation from the gas molecule to phosphorene, whereas negative values indicate electron withdrawal from phosphorene by the gas molecule. The analysis reveals that CO and H_2_S primarily act as electron donors, transferring electrons to the phosphorene substrate. In contrast, SO_2_ behaves as an electron acceptor, withdrawing electrons from the surface. CO_2_ exhibits negligible charge transfer, indicating weak interaction with both the Co-doped and Co-adsorbed phosphorene. The calculated charge transfer values are summarized in Table 2. A comparison of the two modification strategies highlights distinct differences. The Co-adsorbed phosphorene system exhibits significantly higher charge transfer than the Co-doped system. This behavior is consistent with its larger adsorption energies and more pronounced bandgap modulation, suggesting stronger orbital hybridization with gas molecules. However, the strong charge transfer in the Co-adsorbed phosphorene results in very strong binding and near-irreversible adsorption, which limits its suitability for reusable gas sensors. In contrast, Co-doped phosphorene demonstrates moderate charge transfer, offering a favorable balance between interaction strength and reversibility. This ensures detectable electronic responses while maintaining rapid recovery, making Co-doped phosphorene a promising candidate for practical gas-sensing applications.

**Table 2:** Mulliken charge transfer (Δ*Q*) for gas adsorption on the Co-modified phosphorene

Gas	Δ*Q* (Co-doped, e)	Nature	Δ*Q* (Co-adsorbed, e)	Nature
CO	+0.18 to + 0.25	Donor	+0.40 to +0.55	Strong donor
CO_2_	∼±0.05	Weak interaction	+0.15 to +0.25	Moderate donor
H_2_S	+0.08 to +0.12	Weak donor	+0.25 to +0.35	Moderate donor
SO_2_	−0.15 to −0.22	Acceptor	−0.30 to −0.45	Strong acceptor

**Table 3:** Adsorption (*E*_ads_) and bandgap (*E*_g_) energies of gas molecules adsorbed on Co-doped and Co-adsorbed phosphorene

Gas molecule	*E*_ads_ (eV)	*E*_g_ (eV)	Gas molecule	*E*_ads_ (eV)	*E*_g_ (eV)
Co-doped-phosp	—	0.83	Co-adsorbed-phosp	—	0.42
CO	−1.20	0.50	CO	−4.00	0.3
CO_2_	−0.42	0.87	CO_2_	−1.90	0.34
H_2_S	−0.17	0.63	H_2_S	−2.60	0.25
SO_2_	−0.55	0.60	SO_2_	−3.10	0.29

These findings highlight the critical role of Co modification in tuning charge transfer and gas-sensing behavior, providing a theoretical basis for selecting materials with optimal sensitivity and reversibility. Overall, the sensing performance of the two systems shows distinct trends. While Co-adsorbed phosphorene exhibits stronger adsorption energies and larger charge transfer, this leads to excessively strong binding and limited recovery, making it less suitable for reusable sensing applications. Conversely, Co-doped phosphorene demonstrates moderate charge transfer combined with pronounced bandgap modulation, resulting in high electronic sensitivity and faster recovery. Therefore, Co-doped phosphorene is more suitable for practical gas-sensing applications, whereas Co-adsorbed phosphorene may be better suited for gas capture or storage.

### Sensing response (SR)

The sensing response of a gas sensor is defined as the relative change in the electrical conductivity of Co-doped phosphorene and Co-adsorbed phosphorene upon gas adsorption. It is a crucial parameter for evaluating the effectiveness of the sensor in detecting commonly encountered gas species. Determining the sensing response of a material for each gas molecule remains challenging in computational studies. We note that the sensing response estimated from eqn (5) and (6) is based on a simplified Arrhenius-type relation, where bandgap changes are used as a proxy for conductivity.^57^ This approach neglects factors such as carrier concentration, scattering, and transport effects, and therefore provides qualitative trends only. Moreover, explicit transport calculations (*e.g.*, NEGF or I–V characteristics) are not included, so the predicted responses should be interpreted cautiously.5
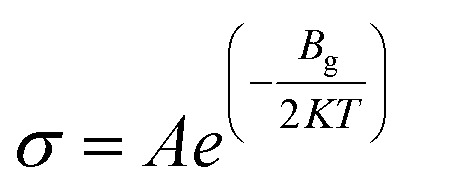
6
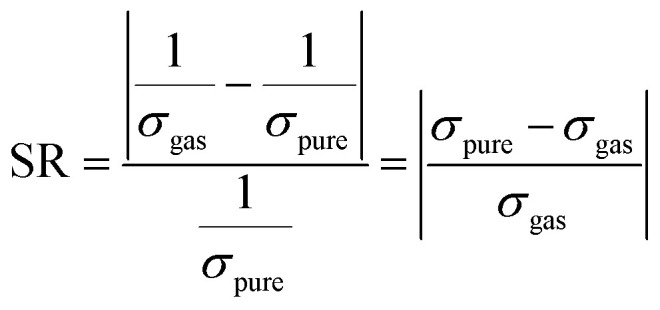


In the above equations, *σ*, *B*_g_, *A*, *T*, and *K* denote the conductivity, bandgap energy, a constant, temperature, and the Boltzmann constant, respectively.

**Fig. 8:**
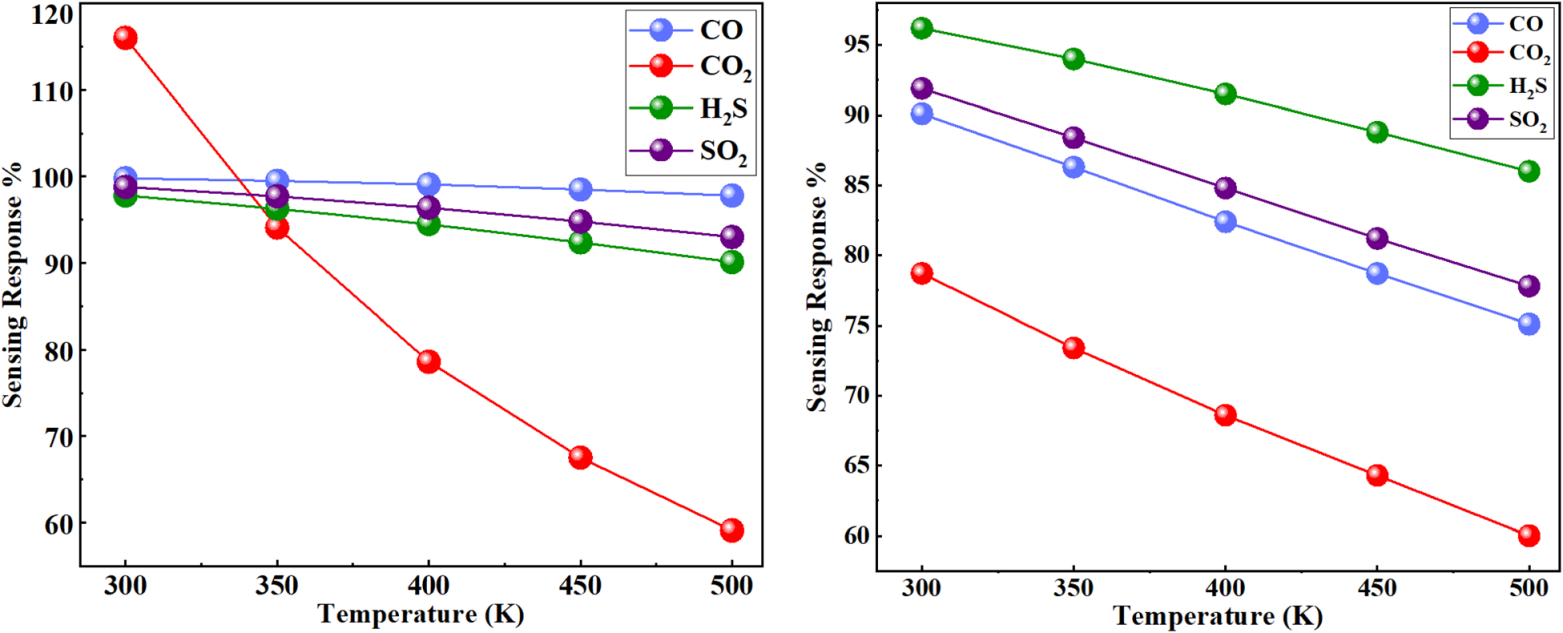
Temperature-dependent sensing responses (%) of the Co-doped and Co-adsorbed phosphorene toward SO_2_, H_2_S, CO_2_, and CO. Co doping shows higher and more stable responses, while all responses decrease with increasing temperature.

The sensing responses of the Co-doped and Co-adsorbed phosphorene toward SO_2_, H_2_S, CO_2_, and CO exhibit a clear dependence on temperature, with noticeable differences between the two configurations. Across all gases and temperature ranges, the Co-doped system shows relatively higher sensing responses than the Co-adsorbed counterpart, suggesting comparatively stronger interactions with the target molecules (Fig. 8). In both systems, the sensing response decreases as the temperature increases from 300 K to 500 K, which can be attributed to enhanced thermal agitation that weakens adsorption strength and reduces charge transfer. Among the gases studied, SO_2_ and CO show the highest responses, whereas CO_2_ exhibits the lowest, particularly in the Co-adsorbed configuration. The difference between doping and adsorption is more evident for CO_2_ and H_2_S, where the doped system retains comparatively higher responses at elevated temperatures. Overall, these results suggest that both modification strategies improve the sensing characteristics of phosphorene, with Co doping showing comparatively better potential under varying thermal conditions. The temperature-dependent sensing responses are presented in Table 4.

**Table 4:** Sensing response of Co-doped and adsorbed phosphorene toward toxic gases at different temperatures

Temp. (K)	Co-doped-phosp				Co-adsorbed-phosp			
	SO_2_	H_2_S	CO_2_	CO	SO_2_	H_2_S	CO_2_	CO
300	−0.988	−0.978	−1.160	−0.998	−0.919	−0.962	−0.787	−0.901
350	−0.977	−0.963	−0.941	−0.995	−0.884	−0.940	−0.734	−0.863
400	−0.964	−0.945	−0.786	−0.991	−0.848	−0.915	−0.686	−0.824
450	−0.948	−0.924	−0.675	−0.985	−0.812	−0.888	−0.643	−0.787
500	−0.930	−0.901	−0.591	−0.978	−0.778	−0.860	−0.604	−0.751

### Recovery time

Recovery times, defined as the period required for gas desorption, were estimated using a simplified Arrhenius-type model with a fixed attempt frequency of 10^12^ s^−1^, a standard approximation in computational studies of gas adsorption.^58,59^ Although this approach neglects surface-specific vibrational modes and dynamic effects, it provides a reasonable first-order estimate of desorption kinetics. Furthermore, visible-light irradiation can enhance desorption by supplying additional energy to overcome adsorption barriers, suggesting that actual recovery times may be shorter than those predicted by the thermal model. The near-irreversible adsorption on the Co-adsorbed surface may indicate potential applications in pollutant capture.

In this work, the recovery time is estimated using a Van't Hoff–Arrhenius-type expression.^60–62^7
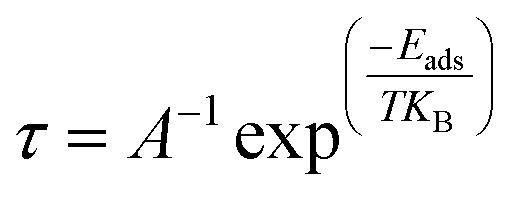
Here, *τ*, *A*, *E*_ads_, *T*, and *K*_B_ represent the recovery time, frequency (10^12^ s^−1^ for visible light), adsorption energy, temperature, and Boltzmann constant, respectively.

It should be noted that this approach is highly simplified. The chosen pre-exponential factor is approximate, and the model neglects important effects, such as multiple desorption pathways, photon-assisted processes, surface defects, and realistic experimental conditions. Therefore, the calculated recovery times should be interpreted only in terms of order-of-magnitude trends, rather than as quantitative predictions. The estimated recovery times are summarized in Table 5. For the Co-doped system, relatively short recovery times are obtained for SO_2_, H_2_S, and CO_2_, indicating that these gases can desorb readily, even at room temperature. In contrast, CO shows comparatively longer recovery times, suggesting stronger binding and the need for elevated temperatures for efficient desorption. These results indicate a balance between adsorption strength and reversibility, which is desirable for sensing applications. In contrast, the Co-adsorbed system exhibits extremely large recovery times across all gases and temperatures. Rather than representing physically meaningful timescales, these values indicate that desorption is effectively negligible under practical conditions. This suggests that gas adsorption on Co-adsorbed phosphorene is essentially irreversible within experimental timescales, limiting its applicability for reusable sensing devices. However, such a strong binding may be advantageous for applications such as gas capture and catalytic processes.

**Table 5:** Estimated recovery times (s) based on the Arrhenius model. Values indicate order-of-magnitude trends rather than precise predictions

Temp. (K)	Co-doped-phosp				Co-adsorbed-phosp			
	SO_2_	H_2_S	CO_2_	CO	SO_2_	H_2_S	CO_2_	CO
300	1.74 × 10^−3^	7.18 × 10^−10^	1.14 × 10^−5^	1.44 × 10^8^	2.51 × 10^38^	4.78 × 10^31^	8.31 × 10^19^	6.91 × 10^51^
350	8.31 × 10^−5^	2.80 × 10^−10^	1.12 × 10^−6^	1.90 × 10^5^	1.58 × 10^31^	2.75 × 10^25^	2.29 × 10^15^	5.24 × 10^42^
400	8.51 × 10^−6^	1.39 × 10^−10^	1.96 × 10^−7^	1.32 × 10^3^	6.31 × 10^25^	5.75 × 10^20^	8.71 × 10^11^	7.58 × 10^35^
450	1.44 × 10^−6^	8.02 × 10^−11^	5.06 × 10^−8^	2.75 × 10^1^	3.98 × 10^21^	1.32 × 10^17^	1.90 × 10^9^	3.63 × 10^30^
500	3.50 × 10^−7^	5.17 × 10^−11^	1.71 × 10^−8^	1.25	1.74 × 10^18^	1.61 × 10^14^	1.42 × 10^7^	2.01 × 10^26^

## Conclusion

In this work, the adsorption and sensing behaviors of toxic gases (CO, CO_2_, H_2_S, and SO_2_) on Co-doped and Co-adsorbed phosphorene were systematically investigated using first-principles density functional theory calculations. Cobalt doping significantly modifies the electronic structure of phosphorene, with moderate charge transfer from gas molecules inducing bandgap changes and enhanced optical absorption, which together contribute to improved and reversible gas-sensing performance. By comparison, excessive charge transfer in the Co-adsorbed system results in stronger adsorption and near-irreversible binding, limiting reusability but indicating potential for gas capture and catalytic applications. Among the studied gases, SO_2_ and CO exhibit the strongest sensing responses in both configurations, while CO_2_ interacts most weakly, particularly on the Co-adsorbed surface. The difference between doping and adsorption is most pronounced for CO_2_ and H_2_S, with the Co-doped system maintaining sensitivity and thermal stability even at elevated temperatures. Recovery-time analysis indicates that Co-doped phosphorene allows efficient desorption under visible-light irradiation, with rapid recovery of SO_2_, H_2_S, and CO_2_ at room temperature and moderate recovery of CO at higher temperatures. By contrast, adsorption on Co-adsorbed phosphorene is effectively irreversible under practical conditions, limiting its suitability for reusable sensing, while highlighting applications in pollutant capture, storage, or catalysis. Overall, Co-doped phosphorene achieves a favorable balance of adsorption strength, tunable electronic response, and recovery efficiency, demonstrating its potential as a versatile platform for toxic gas sensing applications.

## Conflicts of interest

There are no conflicts to declare.

## Data Availability

The data presented in this work are exclusively generated from first-principles simulations performed using the SIESTA package. They do not originate from any external crystallographic database or previously published reference sources. Instead, all results are obtained directly from computational modeling and are reported in the form of figures and tables within the manuscript.
